# Potential role of HTLV-1 Tax-specific cytotoxic t lymphocytes expressing a unique t-cell receptor to promote inflammation of the central nervous system in myelopathy associated with HTLV-1

**DOI:** 10.3389/fimmu.2022.993025

**Published:** 2022-08-23

**Authors:** Yukie Tanaka, Tomoo Sato, Naoko Yagishita, Junji Yamauchi, Natsumi Araya, Satoko Aratani, Katsunori Takahashi, Yasuo Kunitomo, Misako Nagasaka, Yoshinobu Kanda, Kaoru Uchimaru, Tomohiro Morio, Yoshihisa Yamano

**Affiliations:** ^1^ Department of Molecular Microbiology, Tokyo Medical and Dental University (TMDU), Tokyo, Japan; ^2^ Research Core, Institute of Research, Tokyo Medical and Dental University (TMDU), Tokyo, Japan; ^3^ Department of Rare Diseases Research, Institute of Medical Science, St. Marianna University School of Medicine, Kawasaki, Japan; ^4^ Division of Neurology, Department of Internal Medicine, St. Marianna University School of Medicine, Kawasaki, Japan; ^5^ Advanced Business Promotion Department, Business Development Segment, LSI Medience Corporation, Tokyo, Japan; ^6^ Chao Family Comprehensive Cancer Center, University of California Irvine School of Medicine, Orange, CA, United States; ^7^ Division of Hematology, Jichi Medical University Saitama Medical Center, Saitama, Japan; ^8^ Division of Hematology, Department of Medicine, Jichi Medical University, Tochigi, Japan; ^9^ Department of Hematology and Oncology, Research Hospital, The Institute of Medical Science, The University of Tokyo, Tokyo, Japan; ^10^ Laboratory of Tumor Cell Biology, Department of Computational Biology and Medical Sciences, Graduate School of Frontier Sciences, The University of Tokyo, Tokyo, Japan; ^11^ Department of Pediatrics and Developmental Biology, Tokyo Medical and Dental University (TMDU), Tokyo, Japan

**Keywords:** tax, T-cell receptor repertoire, Cytotoxic T-cell, CSF, HAM

## Abstract

Human T-lymphotropic virus 1 (HTLV-1) infection causes two serious diseases: adult T-cell leukemia/lymphoma (ATL) and HTLV-1-associated myelopathy (HAM). Immunological studies have revealed that HTLV-1 Tax-specific CD8^+^ cytotoxic T-cells (Tax-CTLs) in asymptomatic carriers (ACs) and ATL patients play an important role in the elimination of HTLV-1-infected host cells, whereas Tax-CTLs in HAM patients trigger an excessive immune response against HTLV-1-infected host cells infiltrating the central nervous system (CNS), leading to local inflammation. Our previous evaluation of HTLV-1 Tax_301-309_ (SFHSLHLLF)-specific Tax-CTLs (Tax_301-309_-CTLs) revealed that a unique T-cell receptor (TCR) containing amino acid (AA)-sequence motif PDR, was shared among HLA-A*24:02^+^ ACs and ATL patients and behaved as an eliminator by strong activity against HTLV-1. However, it remains unclear whether PDR^+^Tax_301-309_-CTLs also exist in HLA-A*24:02^+^ HAM patients and are involved in the pathogenesis of HAM. In the present study, by high-throughput TCR repertoire analysis technology, we revealed TCR repertoires of Tax_301-309_-CTLs in peripheral blood (PB) of HLA-A*24:02^+^ HAM patients were skewed, and a unique TCR-motif PDR was conserved in HAM patients (10 of 11 cases). The remaining case dominantly expressed (-DR, P-R, and PD-), which differed by one AA from PDR. Overall, TCRs with unique AA-sequence motifs PDR, or (-DR, P-R, and PD-) accounted for a total of 0.3-98.1% of Tax_301-309_-CTLs repertoires of HLA-A*24:02^+^ HAM patients. Moreover, TCR repertoire analysis of T-cells in the cerebrospinal fluid (CSF) from four HAM patients demonstrated the possibility that PDR^+^Tax_301-309_-CTLs and (-DR, P-R, and PD-)^+^Tax_301-309_-CTLs efficiently migrated and accumulated in the CSF of HAM patients fostering increased inflammation, although we observed no clear significant correlation between the frequencies of them in PB and the levels of CSF neopterin, a known disease activity biomarker of HAM. Furthermore, to better understand the potential function of PDR^+^Tax_301-309_-CTLs, we performed immune profiling by single-cell RNA-sequencing of Tax_301-309_-CTLs, and the result showed that PDR^+^Tax_301-309_-CTLs up-regulated the gene expression of natural killer cell marker *KLRB1* (CD161), which may be associated with T-cell activation and highly cytotoxic potential of memory T-cells. These findings indicated that unique and shared PDR^+^Tax_301-309_-CTLs have a potential role in promoting local inflammation within the CNS of HAM patients.

## Introduction

Human T lymphotropic virus 1 (HTLV-1) is a human retrovirus, and most individuals infected with HTLV-1 remain asymptomatic carriers (ACs) throughout their lives (1, 2). However, some infected individuals develop HTLV-1-associated diseases including two major serious diseases, adult T-cell leukemia/lymphoma (ATL) and HTLV-1-associated myelopathy (HAM). ATL is an aggressive mature T-cell malignancy with a poor prognosis that occurs in approximately 5% of HTLV-1-infected individuals (3, 4) and HAM is a chronic inflammatory neurological disease of the central nervous system (CNS) that occurs in approximately 0.25-3.8% of HTLV-1-infected individuals (5–7). Thus, even though ATL and HAM are both HTLV-1-associated diseases, their pathogenesis is quite different, and the corresponding T-cell immune responses against HTLV-1 lead to distinct beneficial and detrimental contributions in their pathogenesis (7–10).

Tax, a regulatory protein of HTLV-1, is not only involved in viral transcription but is also known to be the major target antigen for HTLV-1-specific CD8^+^ cytotoxic T-cells (CTLs). Accordingly, HTLV-1 Tax-specific CTLs (Tax-CTLs) act as a pivotal mediator that eliminates infected host cells (11, 12). In our previous studies on the T-cell receptor (TCR) of HLA-A*24:02-restricted Tax_301–309_ (SFHSLHLLF)-specific CTLs (Tax_301-309_-CTLs), we found that a unique amino acid (AA)-sequence motif, PDR in the complementarity-determining region 3 (CDR3) of TCR-β chain was shared among ACs and ATL patients undergoing allogeneic hematopoietic stem cell transplantation (allo-HSCT) (13, 14). Tax_301-309_-CTLs expressing PDR-motif (PDR^+^Tax_301-309_-CTLs) were often predominantly observed in peripheral blood (PB) of HLA-A*24:02^+^ ACs and well-controlled ATL long-term survivors after allo-HSCT and exerted strong and selective cytotoxicity against HTLV-1-infected cells *in vitro* (13–16). These results suggested that PDR^+^Tax_301-309_-CTLs, which have strong activity against HTLV-1 might play an important role in reducing the risk of the onset of ATL during the AC phase and in preventing relapse of ATL patients after allo-HSCT.

On the other hand, the pathogenesis of HAM is thought to be triggered by an excessive T-cell immune response, centered on Tax-CTLs, against HTLV-1-infected cells infiltrating the CNS, resulting in damage to CNS resident cells, described as “bystander damage” (8, 17, 18). So far, TCR repertoire analysis of Tax-CTLs in HAM patients, especially HLA-A*24:02^+^ patients, has not been adequately carried out, and it is unclear how Tax-CTLs could be involved in CNS inflammation. Therefore, we hypothesized that if HLA-A*24:02^+^ HAM patients, as well as ACs and ATL patients, share very high cytotoxic PDR^+^Tax_301-309_-CTLs, this may infiltrate the CNS and detrimentally contribute to HTLV-1-specific inflammatory responses, ultimately affecting the morbidity and severity of HAM. Although several studies have reported the accumulation of Tax-CTLs in the cerebrospinal fluid (CSF) of HAM patients (19, 20), none have focused on the potential role of a unique CTL clonal component of Tax-CTLs, such as PDR^+^Tax_301-309_-CTLs, in promoting local inflammation within the CNS of HAM patients.

In this study, we comprehensively evaluated the TCR repertoires of Tax_301-309_-CTLs in both PB and CSF of HLA-A*24:02^+^ HAM patients to better understand the potential role of shared PDR^+^Tax_301-309_-CTLs in promoting the inflammatory pathogenesis of HAM.

## Materials and methods

### Cells

For all experiments, the used samples were from HLA-A*24:02^+^ individuals. PB from fifteen HAM patients and CSF from four HAM patients were collected at St. Marianna University School of Medicine, respectively. PB samples of twelve ACs were collected at the Institute of Medical Science, The University of Tokyo Hospital. Patients with HAM were diagnosed based on the World Health Organization (WHO) guidelines (21), and the clinical information has been summarized in **Table 1**. The protocol in this study was approved by the Institutional Review Boards of St. Marianna University School of Medicine (#1646), the Institute of Medical Science, The University of Tokyo (30-4-B0501), and Tokyo Medical and Dental University (TMDU) (#O2018-002). All subjects provided written informed consent. Peripheral blood mononuclear cells (PBMCs) were separated by Ficoll-based density gradient centrifugation, and all samples were cryopreserved in liquid nitrogen until use.

**Table:** Table 1 Clinical characteristics of patients with HAM and ACs enrolled in this study.

Patient ID	Age (years)	Sex	HLA-A		Disease duration	used sample	WBC ( /µl)	Lymphocytes (%)	PVL /100 PBMCs	CSF neopterin (pmol/mL)	CSF CXCL10 (pg/ml)	Steroid therapy
HAM-1	77	F	A*02:01	A*24:02	18 years	PBMCs	6350	18.6	3.0	6	414.9	-
HAM-2	60	M	A*11:01	A*24:02	33 years	PBMCs	10100	15.5	4.0	18	5006.6	+
HAM-3	65	M	A*24:02	A*26:03	20 years	PBMCs	6100	40.8	8.9	7	672.1	+
HAM-4	68	F	A*11:01	A*24:02	17 years	PBMCs	10800	13.0	2.9	4	814.2	+
HAM-5	77	F	A*02:06	A*24:02	11 years	PBMCs	7320	16.7	2.2	14	2197.0	+
HAM-6	75	F	A*11:01	A*24:02	16 years	PBMCs	7120	14.9	3.2	27	4598.1	+
HAM-7	77	F	A*24:02	A*31:01	20 years	PBMCs	9200	22.1	6.0	38	4279.6	+
HAM-8	81	M	A*24:02	A*31:01	13 years	PBMCs/CSF	7520	31.7	21.3	18	3690.9	+
HAM-9	70	F	A*24:02	A*24:02	9 years	PBMCs/CSF	8300	21.7	8.8	35	3825.7	+
HAM-10	63	F	A*24:02	A*31:01	29 years	PBMCs	6230	42.5	1.3	4	641.7	+
HAM-11	39	F	A*24:02	A*33:03	8 years	PBMCs/CSF	6600	24	2.1	31	6187.5	+
HAM-12	56	F	A*24:02	A*24:02	4 years	PBMCs/CSF	4900	28.5	3.8	17	3216.7	+
HAM-13	38	F	A*24:02	A*24:02	6 years	PBMCs	7900	30	2.1	11	2136.5	+
HAM-14	50	F	A*24:02	A*24:02	7 years	PBMCs	5000	36.1	13.1	38	17120.9	+
HAM-15	53	F	A*11:01	A*24:02	6 years	PBMCs	3900	27.4	6.5	19	2842.8	-
ACs	58 (46-70)	F/M	A*24:02			PBMCs	580 (4330-9210)	32.2 (14.0-38.5)	3.1 (0.1-19.3)			

Fifteen HLA-A*24:02-positive HAM patients between the ages of 38 and 81 years and twelve asymptomatic carriers (ACs) were enrolled in this study. The age and PVL values of ACs show the mean values (ranges). ID, identifier; F, female; M, male; CSF, cerebrospinal fluid; PVL, HTLV-1 proviral copies/100 PBMCs; CXCL10, C-X-C motif chemokine 10; Steroid therapy, oral steroid therapy with prednisolone.

### Measurement of HTLV-1 proviral load and CSF biomarkers

PVL in PBMCs was measured using real-time quantitative PCR targeting HTLV-1 tax, as a previous report (22), and compensated using standard reference material (23). CSF level of CXC motif chemokine 10 (CXCL10) was measured using a cytometric bead array (CBA, BD Biosciences, San Jose, CA) and CSF neopterin level was commercially measured using high-performance liquid chromatography (SRL Inc., Tokyo, Japan).

### Multi-color flow cytometry and sorting

Thawed PBMCs were reacted with LIVE/DEAD Fixable Aqua Dead Cell Stain Kit (Thermo Fisher Scientific, Waltham, MA, USA) to remove the dead cells. For phenotypic analysis, cells were stained with phycoerythrin (PE)-conjugated Tax_301-309_/HLA-A*24:02 tetramer reagents (MBL, Nagoya, Japan) and several fluorescence-conjugate mouse anti-human monoclonal antibodies (mAbs) [CD3-APC-H7, CD8-Pacific Blue, CD45RA-PerCP5.5, CCR7-Alexa647, CD62L-PE-Cy7, CD27-FITC, CXCR3-BV605 (BD Biosciences), and CD95-PE-Cy5 (Biolegend, San Jose, CA)] for 25 min on ice. Stained cells were washed twice and immediately acquired using FACSAriaIII Fusion (BD Biosciences) equipped with 20 detectors by 4-lasers at 488 nm, 561 nm, 633 nm, and 405 nm. The data were analyzed using FlowJo ver.10 software (BD Biosciences). The experiments requiring cell sorting for TCR repertoire analysis, described below, were carried out using the same equipment.

### TCR repertoire analysis by next-generation sequencing

TCR repertoires of FACS-sorted Tax_301-309_-CTLs (approximately 0.5-8.5 x10^4^ cells) and CD8^+^ T-cells (approximately 1.5-6.3 x10^5^ cells) in PBMCs from eleven HAM patients (HAM-1, -4, -5, -7, -8, -9, -11, -12, -13, -14, and -15) and CSF whole cells (approximately 0.8-2.7 x 10^4^ cells) of four HAM patients (HAM-8, -9, -11, and -12) were analyzed. The total RNA of each sample was independently extracted using the RNeasy Micro kit (Qiagen, Valencia, CA). Then, cDNA was amplified using iRepertoire human TCRβ kits (iRepertoire, Huntsville, AL, USA) according to the manufacturer’s protocol. The quality (size and integrity) and quantity (concentration) of the final library for sequencing were checked by the TapeStation4150 system (Agilent Technologies, Santa Clara, USA) and Qubit 4.0 fluorometer (Thermo Fisher Scientific), respectively. Sequencing was performed using MiSeq platform (Illumina, San Diego, CA, USA) with 250 bp paired-end reads. The data were analyzed in a provided pipeline by iRepertoire (http://www.irepertoire.com). The illustrative tree map was used to represent each unique T-cell clone. The sequence run data including reads, total CDR3, and distinct CDR3 have been summarized in **Supplementary Table 1**.

### Single-cell RNA-sequencing for Tax_301-309_-CTLs

scRNA sequencing for FACS-sorted Tax_301-309_-CTLs in PBMCs from three HAM patients were performed using the microwell-based BD Rhapsody Single-Cell Analysis System (BD Biosciences). Cell lysis, cDNA synthesis, and library construction were performed according to the manufacturer’s protocols (24). Briefly, approximately 1.0 x 10^3^ (HAM-1), 5.1 x 10^4^ (HAM-7), and 4.3 x 10^3^ (HAM-8) live Tax_301-309_-CTLs were sorted by FACSAriaIII Fusion, centrifuged, and resuspended in cold sample buffer, respectively. Following viability confirmation (>92%), each cell sample was independently loaded on a Rhapsody Cartridge for single-cell capture and cDNA library preparation using the BD Rhapsody Express System (BD Biosciences). In the process, estimated 543 cells (HAM-1), 13,057 cells (HAM-7), and 2,053 cells (HAM-8) were captured by cell capture beads, respectively. Following single-cell capture, we performed cDNA library construction for VDJ TCR, sample tags, and the targeted mRNA (259 different genes) with Human T-Cell Expression Panel, according to the manufacturer’s protocols. Size selection was performed using AMPure XP magnetic beads (Beckman Coulter, Brea, CA, USA). The quality and quantity checks of the library were assessed using Agilent4150 TapeStation system and Qubit 4.0 fluorometer, respectively. Finally, prepared sequence libraries from all three patients were pooled together in a ratio of 1 (targeted mRNA 2000 reads/cell): 5.5 (VDJ TCR 3000 reads/cell) and commercially sequenced on Illumina NextSeq500 with paired-end reads (75-bp for Read 1 and 225-bp for Read 2) by Macrogen (Seoul, South Korea).

### scRNA-seq data processing and analysis

FASTQ sequence data files were processed on Seven Bridges Genomics online platform (https://www.sevenbridges.com) by running the BD Rhapsody Targeted Analysis Pipeline with V(D)J processing incorporated, following the company’s instructions.

After identifying the cell barcode and the unique molecular index (UMI), recursive substitution error correction (RSEC) counts as the final molecular counts by removing the effect of UMI errors were calculated. Quality control for removing dead cells was adopted using the putative cell detection function in the Seven Bridge pipeline as the first step, and then we excluded cell based on the distribution of gene and transcript counts as the following quality criteria: less than 25 expressed genes and less than 50 detected transcripts. RSEC counts were used for downstream analysis with SeqGeq version 1.7.0 (BD Biosciences) and R version 4.0.2. After RSEC data files were concatenated together, the plug-in Lex-BDSMK was run to separate the sample tags, then the plug-in VDJ Explorer to identify individual TCR CDR3 sequences. Consequently, a total of 11,029 TCR paired with mRNA expression were successfully assembled from the three patients’ data. Then, we sorted the unique CDR3-AA PDR-motif and (PD-, P-R, and -DR)-motif expressing TCR clones also by plugin-VDJ Explorer, and the data was concatenated and supplied to further process in differentially expressed gene (DEG) analysis. Furthermore, the data of 11,029 TCRs of Tax_301-309_-CTL clones sorted with PDR-motif expressing TCR clones were also proceeded in Seurat (version 4.0.1) package to perform downstream cell clustering. For cell clustering, principal component analysis (PCA) was performed to determine the number of clusters, and UMAP for two-dimensional data visualization using PCA data was conducted. GO (Gene ontology) function annotation and pathway enrichment analysis of the target genes were performed using the Metascape database platform (https://metascape.org/gp/index.html#/main/step1).

### Statistical analysis

Statistical analyses were performed using GraphPad Prism 9 (GraphPad Software Inc., San Diego, CA). Differences in the frequencies and the differentiation subsets of Tax_301-309_-CTLs between ACs and HAM patients were tested using the Mann-Whitney U-test. Correlation between the CSF markers (CXCL10 and neopterin) and the frequencies of Tax_301-309_-CTLs expressing (PDR, -DR, P-R, and PD-)-motifs in PB were tested by Spearman’s rank correlation test. *P*-values, 0.05 were considered statistically significant. In the scRNA-seq experiments, DEG analysis expressing fold change was performed using Bonferroni adjusted *p*< 0.05 relative to comparator populations.

## Results

### Frequencies and differentiation of Tax_301-309_-CTLs in HAM patients

**Figure 1:**
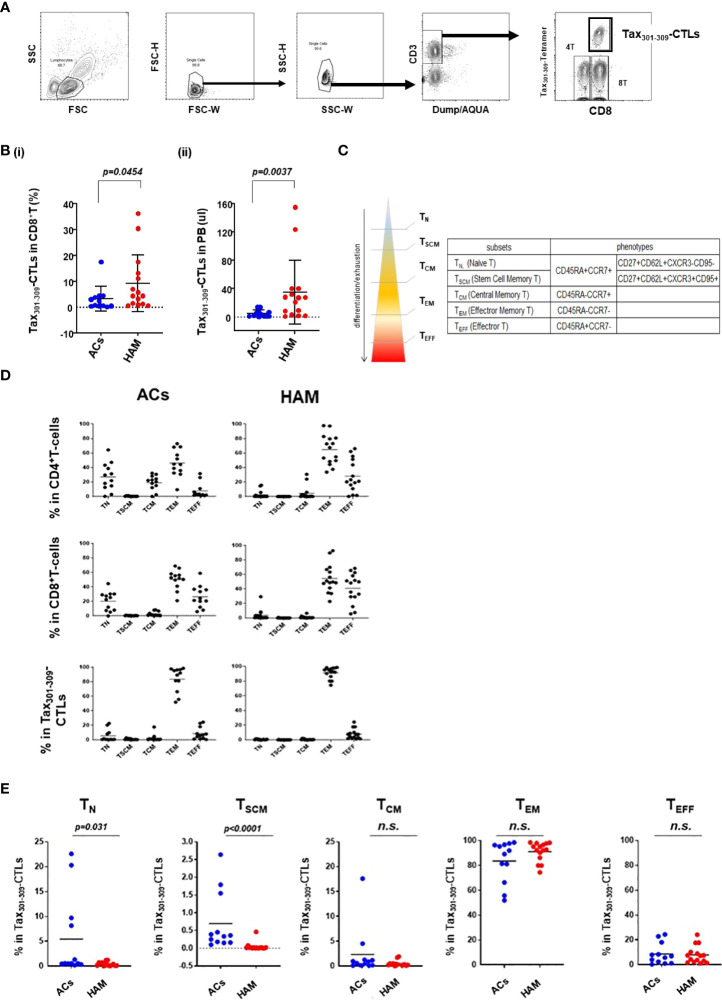
The frequencies and differentiation status of Tax_301-309_-CTLs in PBMCs of HAM patients and ACs **(A)** gating strategy to define live CD4^+^ T-cells, CD8^+^ T-cells and Tax_301-309_-CTLs by ten-color flowcytometry. **(B)** comparison of the frequencies of Tax_301-309_-CTLs (i) in CD8^+^ T-cells (%) and (ii) the absolute frequencies of Tax_301-309_-CTLs in PB between ACs and HAM patients. **(C)** the hierarchy model of five T-cell differentiation subsets (T_N_, T_SCM_, T_CM_, T_EM_, and T_EFF_) and the corresponding phenotypes. **(D)** the percentage of the five T-cell differentiation subsets in CD4^+^ T-cells, CD8^+^ T-cells, and Tax_301-309_-CTLs of HAM patients and ACs. **(E)** comparison of the percentages of Tax_301-309_-CTLs in the five T-cell differentiation subsets between ACs and HAM patients, respectively. *p* values were calculated using the Mann-Whitney U test. n.s., no significant.

The frequencies and differentiation status of Tax_301-309_-CTLs in PBMCs of HAM patients were evaluated compared with those of ACs (**Figure 1** and **Table 2**). **Figure 1A** shows a detection panel of each population of live- CD4^+^ T-cells, CD8^+^ T-cells, and Tax_301-309_-CTLs in PBMCs by 10-color flowcytometry. The percentage of Tax_301-309_-CTLs in CD8^+^ T-cells and the absolute frequencies of Tax_301-309_-CTLs in PBMCs from HAM patients were significantly higher than those of ACs (**Figure 1B** **-i** and **-ii**, respectively), which results were consistent with previous reports (19, 25, 26).

**Table:** Table 2 Tax_301-309_-CTL profiles of HLA-A*24:02^+^ HAM patients and ACs.

Patient ID	Tax_301-309_-CTLs in PB		T-cell differentiation status of Tax_301-309_-CTLs (%)				
	(% in 8T)	( /µl)	T_N_	T_SCM_	T_CM_	T_EM_	T_EFF_
HAM-1	1.0	1.1	0.2	0.08	0.2	92.1	7.5
HAM-2	1.3	3.0	0.7	UD	0.0	90.4	8.9
HAM-3	4.4	28.1	0.1	UD	0.06	92.7	7.2
HAM-4	13.2	27.7	0.2	0.02	0.04	98.3	1.4
HAM-5	0.6	1.7	1.2	0.5	0.5	86.2	10.2
HAM-6	0.6	1.6	1.2	UD	0.2	74.4	24.2
HAM-7	17.5	123.5	0.02	UD	0.3	96.6	3.1
HAM-8	11.2	39.9	0.63	0.01	1.9	79.9	17.6
HAM-9	36.3	155.0	0.0	0.02	0.36	98.5	1.1
HAM-10	3.2	8.3	0.4	0.02	0.0	95.2	4.4
HAM-11	1.5	9.2	0.70	0.01	0.5	95.1	3.8
HAM-12	4.4	21.0	0.1	0.02	0.3	97.9	1.7
HAM-13	7.0	31.9	0.1	0.02	0.2	92.2	7.5
HAM-14	5.8	27.3	0.6	0.01	1.7	80.0	17.7
HAM-15	30.5	40.2	0.03	UD	0.2	97.7	2.2
mean ± (SD)	9.2 ± 11.1	34.6 ± 45.1	0.4 ± 0.4*	0.04 ± 0.1*	0.4 ± 0.6*	91.1 ± 7.6**	7.9 ± 6.9**
ACs (n=12)	3.2 ± 4.8	4.6 ± 4.9	5.4 ± 8.2	0.7 ± 0.8	2.3 ± 5.0	83.5 ± 16.8	8.6 ± 8.5

T-cells have been phenotypically divided into the five T-cell differentiation subsets mainly based on CD45RA and CCR7 expression: CD45RA+CCR7+ (T naive [T_N_]), CD45RA-CCR7+ (T central memory [T_CM_]), CCR7-CD45RA- (T effector memory [T_EM_]), and CCR7-CD45RA+(T effector [T_EFF_]) (27) and stem cell memory [T_SCM_], a novel T-cell differentiation subset, with additional other molecule (CD27, CD62L, CXCR3, and CD95) expression in the conventional CD45RA+CCR7+ T_N_ population (28-30), summarized in **Figure 1C**. Each value of ACs shows means ± SD. UD, under detectable. *, *P* < 0.05.

Recently, human T-cells have been phenotypically divided into the five T-cell differentiation subsets mainly based on CD45RA/CCR7 and CD95 molecule expression: CD45RA^+^CCR7^+^ (T naive [T_N_]), CD45RA^-^CCR7^+^ (T central memory [T_CM_]), CCR7^-^CD45RA^-^ (T effector memory [T_EM_]), and CCR7^-^CD45RA^+^(T effector [T_EFF_]) (27), and stem cell memory [T_SCM_], a novel T-cell differentiation subset, mainly expressing CD95 in the conventional CD45RA^+^CCR7^+^ T_N_ population (**Figure 1C**) (28–30). T_SCM_ has properties of differentiated cells yet retain high stemness and phenotypical proximity to naïve cells, therefore, T_SCM_ is understood to be an essential component of the T-cell population for the maintenance of functional immunity in infectious diseases (29).

Tax_301-309_-CTLs in PBMCs of HAM patients showed a clear dominance of T_EM_ (91.1%) among the five T-cell differentiation subsets as well as CD4^+^ T-cells and CD8^+^ T-cells, and the result was comparable to that of ACs (83.5%) (**Figure 1D**). Furthermore, as shown in **Figure 1E**, Tax_301-309_-CTLs of HAM had significantly reduced percentages of each subset of T_N_ and T_SCM_ compared to those of ACs, respectively. In particular, the frequency of Tax_301-309_-CTLs belonging to the T_SCM_ subset of HAM patients were extremely low and undetectable in 5 of 15 cases by our 10-color detection panel for T_SCM_ with CD27^+^CD62L^+^CXCR3^+^CD95^+^ in the conventional T_N_ population.

### Skewed TCR repertoires of Tax_301-309_-CTLs in PBMCs of HLA-A*24:02^+^ HAM patients with a preference for unique sequences

**Figure 2:**
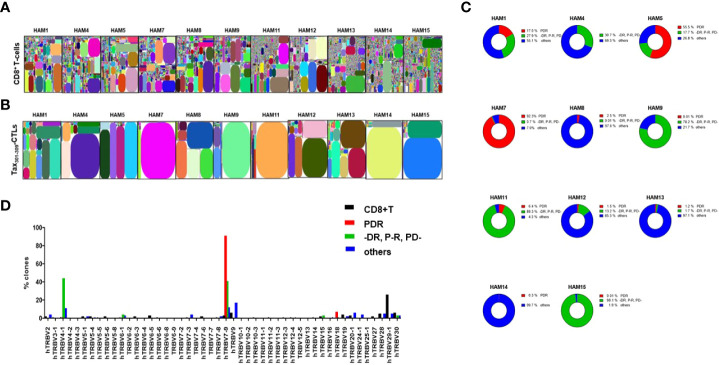
TCR repertoires of CD8^+^ T-cells and Tax_301-309_-CTLs in PBMCs of HAM patients analyzed by a high-throughput sequencing system The illustrative tree maps of TCR repertoires of **(A)** CD8^+^ T-cells and **(B)** Tax_301-309_-CTLs in the PBMCs of HAM patients were generated using iRweb tools (iRepertoire), respectively. Each rectangle plot in the tree map represents a unique T-cell clonotype determined by TCR-ß CDR3 sequences and the size reflects the frequency of each clone. **(C)** ratios of each TCR repertoire type according to the CDR3 AA-sequence motifs (i) PDR, (ii) -DR, P-R, and PD-, and (iii) others to the total number of detected TCR repertoires in Tax_301-309_-CTLs of each HAM patient. **(D)** TCR-BV gene usages of CD8^+^ T-cell clones and Tax_301-309_-CTL clones expressing three types of CDR3 AA-sequences. For CD8^+^ T-cell clones, the TCR-BV gene usage was analyzed within the top 2000 TCR repertoires identified in each patient’s sample.

TCR repertoire analysis of whole CD8^+^ T-cells and Tax_301-309_-CTLs (the sorting gate as shown in **Figure 1A**) in PBMCs of eleven randomly selected HLA-A*24:02^+^ HAM patients were performed with NGS illumina Miseq (**Figure 2**). The TCR-β CDR3 AA-sequence information was summarized in **Supplementary Table 2**. The illustrative tree maps of the whole CD8^+^ T-cell repertoires in PBMCs from HAM patients showed a very wide diversity, with limited clonal expansion of CD8^+^ T-cells (**Figure 2A**). In contrast, Tax_301-309_-CTL repertoires were skewed in all cases analyzed (**Figure 2B**). As expected, PDR, a unique AA-sequence motif in the Tax_301-309_-CTL repertoires, was observed in ten of eleven HLA-A*24:02^+^ HAM patients (0.01-92.3% of Tax_301-309_-CTL repertoires of each patient analyzed) as well as HLA-A*24:02^+^ ACs and ATL patients, previously analyzed (13, 14). In the case (HAM-4) without detection of PDR^+^TCRs, Tax_301-309_-CTL repertoires expressing TCR AA-motif (-DR, P-R, and PD-), which differed by one AA from PDR with the hyphens indicating other AA at these positions, were often observed. In fact, Tax_301-309_-CTL repertoires expressing TCR AA-motif (-DR, P-R, and PD-) have been very frequently observed in not only other HAM patients analyzed in this study but also in ACs and ATL patients in our previous studies (13, 14).

Then, we classified a total of 2,200 Tax_301-309_-CTL clonotypes from eleven HAM patients detected in this experiment into three groups based on their CDR3 AA-sequences with 1) PDR^+^TCRs, 2) (-DR, P-R, and PD-) ^+^TCRs, and 3) others that had no common unique AA-sequence motif. The ratio of each of the three groups based on the AA-sequences to the total TCR repertoires of Tax_301-309_-CTLs in each patient has been summarized in **Figure 2C**. Overall, Tax_301-309_-CTLs expressing TCRs with a unique AA-sequence motif PDR or (DR, P-R, and PD-), accounted for 0.3-98.1% of Tax_301-309_-CTL repertoires in HAM patients. Furthermore, TCR BV gene usage of PDR^+^Tax_301-309_-CTL clones was skewed in favor of the BV7-9 gene and that of (-DR, P-R, and PD-) ^+^Tax_301-309_-CTL clones was skewed in favor of the BV4-1 and BV7-9 genes, while Tax_301-309_-CTLs expressing other TCRs showed variable BV gene usages (**Figure 2D**).

### Accumulation of Tax_301-309_-CTLs in the CSF of HAM patients

**Figure 3:**
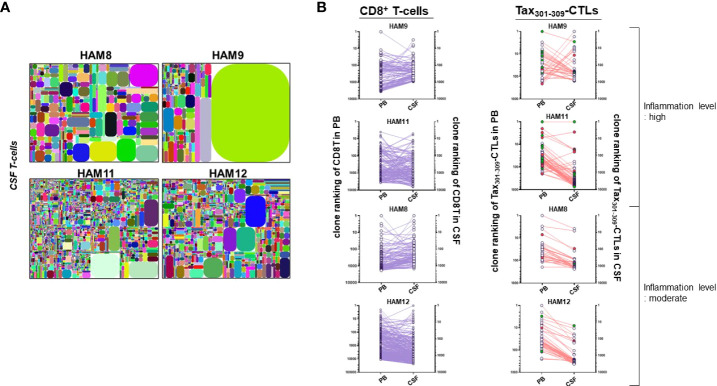
TCR repertoires of whole T-cells in the CSF of HAM patients **(A)** the illustrative tree maps of TCR repertoires of whole T-cells in the CSF from four HAM patients (HAM-8, -9, -11, and -12). **(B)** the clonal rankings of individual CD8^+^ T-cell clones and Tax_301-309_-CTL clones identified in both PB and CSF of four HAM patients. Two HAM patients (HAM-9 and -11) had high levels of CSF neopterin and two HAM patients (HAM-8 and -12) had moderate levels of CSF neopterin. The red circle indicates a PDR^+^Tax_301-309_-CTL clone and the green circle indicates a (-DR, P-R, and PD-)^+^Tax_301-309_-CTL clone.

TCR repertoire analysis of whole T-cells in the CSF of four HLA-A*24:02^+^ HAM patients (HAM-8, -9, -11, and -12) was performed with NGS illumina Miseq (**Figure 3**).

We identified a total of 1,428 (HAM-8), 906 (HAM-9), 6,207 (HAM-11), and 3,002 (HAM-12) T-cell clones in the CSF, respectively (**Supplementary Table 1**). Paired TCR repertoire analysis using PB and CSF samples from the same patients allowed us to identify CD8^+^ T cell and Tax_301-309_-CTL clones infiltrating from PB to CSF. Therefore, we were able to list the top 30 T-cell repertoires in the CSF of four HAM patients, along with the origin of the TCRs of the CD8^+^ T-cells or Tax_301-309_-CTLs (**Table 3**). As shown in **Figure 3A**, the CSF T-cell repertoires of three of four cases (HAM-8, -11, and -12) exhibited very wide clonal diversity, with the most predominant T-cell clone constituting approximately 5.3% of CSF T-cells (**Table 3**). In contrast, the CSF T-cell TCR repertoires of HAM-9 were constituted by a single T-cell clone (approximately 62% of CSF T-cells). This clone was identified as an infiltrating Tax_301-309_-CTL clone from PB.

**Table:** Table 3 TCRß CDR3 amino acid sequences and frequencies of T-cell clones in the CSF of HLA-A*24:02^+^ HAM patients.

Patient / CSF neopterin (pmol/ml)	in CSF					in PB		Patient / CSF neopterin (pmol/ml)	in CSF					in PB	
	clone ranking	CDR3 AA	TRBV	TRBJ	(%)	TCR	clone ranking in CD8^+^ T-cells or Tax_301-309_-CTLs		clone ranking	CDR3 AA	TRBV	TRBJ	(%)	TCR	clone ranking in CD8^+^ T-cells or Tax_301-309_-CTLs
**HAM-9/CSF neopterin** 35	1	ASSVRGNEQF	hTRBV9	hTRBJ2-1	61.7	Tax-CTL	45	**HAM-11/CSF neopterin** 31	1	ASS **P** N **R** AVEQF	hTRBV7-9	hTRBJ2-1	5.7	Tax-CTL	1
	2	ASSVRGAAQF	hTRBV9	hTRBJ2-1	5.9	Tax-CTL	80	2	SVGLQGARGEQY	hTRBV29-1	hTRBJ2-7	3.8	UI	
	3	ASSVRGSPLH	hTRBV9	hTRBJ1-6	2.7	CD8T	2396	3	ASSVRGNEQF	hTRBV9	hTRBJ2-1	3.0	UI	
	4	ASSQ **DR** GFYFGYT	hTRBV4-1	hTRBJ1-2	2.0	Tax-CTL	1	4	ASS **PDR** EQTQY	hTRBV7-9	hTRBJ2-5	2.2	Tax-CTL	5
	5	ASSFYRGPYYNEQF	hTRBV5-6	hTRBJ2-1	1.0	UI		5	ASSPDINYGYT	hTRBV6-5	hTRBJ1-2	0.6	CD8T	56
	6	AWSENTEAF	hTRBV30	hTRBJ1-1	1.0	CD8T	179	6	ASSYSRGGRDEQF	hTRBV6-3	hTRBJ2-1	0.6	CD8T	47
	7	ASRTSGTSDTQY	hTRBV19	hTRBJ2-3	0.9	CD8T	211	7	SVAGNNEQF	hTRBV29-1	hTRBJ2-1	0.6	UI	
	8	AWSSSSTDTQY	hTRBV30	hTRBJ2-3	0.8	Tax-CTL	163	8	SVANTQNTEAF	hTRBV29-1	hTRBJ1-1	0.6	UI	
	9	ASSNTGTGNTGELF	hTRBV7-9	hTRBJ2-2	0.8	Tax-CTL	143	9	ASSVRGAAQF	hTRBV9	hTRBJ2-1	0.6	UI	
	10	SVEAGELF	hTRBV29-1	hTRBJ2-2	0.7	UI		10	ASRNPSGGTDTQY	hTRBV6-1	hTRBJ2-3	0.5	UI	
	11	ASSVGGNEQF	hTRBV9	hTRBJ2-1	0.6	Tax-CTL	174	11	AWTRGEDNEQF	hTRBV30	hTRBJ2-1	0.5	UI	
	12	ASSVKGNEQF	hTRBV9	hTRBJ2-1	0.6	UI		12	ASSGRGITDTQY	hTRBV9	hTRBJ2-3	0.5	CD8T	1972
	13	ASSVRGSEQF	hTRBV9	hTRBJ2-1	0.6	Tax-CTL	134	13	ATSRGLYTDTQY	hTRBV15	hTRBJ2-3	0.4	CD8T	2533
	14	SVESVREAF	hTRBV29-1	hTRBJ1-1	0.5	UI		14	SVRRGSYEQY	hTRBV29-1	hTRBJ2-7	0.4	CD8T	4
	15	ASSVRGTPLH	hTRBV9	hTRBJ1-6	0.5	Tax-CTL	66	15	ASS **P** N **R** QHTQY	hTRBV7-9	hTRBJ2-3	0.4	CD8T	65
	16	ASSSAGVTGELF	hTRBV7-6	hTRBJ2-2	0.5	UI		16	SARERLTGARGGYT	hTRBV20-1	hTRBJ1-2	0.4	CD8T	85
	17	ASSVGADVQPQH	hTRBV9	hTRBJ1-5	0.5	UI		17	ASSAGTSGRAADTQY	hTRBV7-2	hTRBJ2-3	0.4	UI	
	18	AWSPISYNEQF	hTRBV30	hTRBJ2-1	0.5	UI		18	AWSVDSNYGYT	hTRBV30	hTRBJ1-2	0.4	UI	
	19	ASSLPSGGNTDTQY	hTRBV7-6	hTRBJ2-3	0.4	CD8T	1	19	AWSSSSTDTQY	hTRBV30	hTRBJ2-3	0.4	UI	
	20	AWSQGGRGYT	hTRBV30	hTRBJ1-2	0.4	UI		20	AWRDSPYEQY	hTRBV30	hTRBJ2-7	0.3	CD8T	1416
	21	ASSSGVNTEAF	hTRBV5-6	hTRBJ1-1	0.4	UI		21	SVGQGNSYEQY	hTRBV29-1	hTRBJ2-7	0.3	UI	
	22	ASSSRTSGTKNEQF	hTRBV9	hTRBJ2-1	0.3	CD8T	76	22	SVETGESSYEQY	hTRBV29-1	hTRBJ2-7	0.3	UI	
	23	AWTVALTLGYGYT	hTRBV30	hTRBJ1-2	0.3	UI		23	ASSDGYYGYT	hTRBV6-3	hTRBJ1-2	0.3	UI	
	24	SVDGVSTGNEQF	hTRBV29-1	hTRBJ2-1	0.3	UI		24	SIAHTETQY	hTRBV29-1	hTRBJ2-5	0.3	UI	
	25	ACKGGYGYT	hTRBV30	hTRBJ1-2	0.3	UI		25	SVGRDRDEQY	hTRBV29-1	hTRBJ2-7	0.3	UI	
	26	ASRQGNQPQH	hTRBV19	hTRBJ1-5	0.3	UI		26	AWKTVYNEQF	hTRBV30	hTRBJ2-1	0.3	UI	
	27	ASSRNRGEQF	hTRBV7-6	hTRBJ2-1	0.3	UI		27	AWSATSDSGWH	hTRBV30	hTRBJ1-5	0.3	UI	
	28	ASSFVSGARDGYT	hTRBV5-6	hTRBJ1-2	0.3	UI		28	ASGHLLQETQY	hTRBV6-1	hTRBJ2-5	0.3	UI	
	29	ASSARGAAQF	hTRBV9	hTRBJ2-1	0.3	UI		29	AWSRGGTGRST	hTRBV30	hTRBJ1-2	0.3	UI	
	30	ASS **PDR** EETQY	hTRBV7-9	hTRBJ2-5	0.3	Tax-CTL	208	30	ASSLGKDGYT	hTRBV5-1	hTRBJ1-2	0.3	CD8T	117
**HAM-8/CSF neopterin** 18	1	ASSFLLLDEQY	TRBV5-4	TRBJ2-7	5.1	CD8T	491	**HAM-12/CSF neopterin** 17	1	ASAGRYTYEQY	TRBV4-2	TRBJ2-7	5.1	CD8T	13
	2	ASSAGEGNSPLH	TRBV9	TRBJ1-6	4.4	CD8T	13	2	ASSPGTNYGYT	TRBV25-1	TRBJ1-2	3.7	CD8T	4543
	3	SGKQGEGGYT	TRBV29-1	TRBJ1-2	3.5	CD8T	79	3	ASSGSGISTGELF	TRBV7-8	TRBJ2-2	3.1	CD8T	251
	4	SSRPSGDEQF	TRBV29-1	TRBJ2-1	2.9	UI		4	ASSIGTNYGYT	TRBV25-1	TRBJ1-2	2.4	CD8T	278
	5	ASSEMGGADYEQY	TRBV6-1	TRBJ2-7	2.4	CD8T	363	5	SVQGGAVNTEAF	TRBV29-1	TRBJ1-1	1.5	CD8T	675
	6	ASSVRGNEQF	TRBV9	TRBJ2-1	2.3	Tax-CTL	1	6	ASSSPGTGDQETQY	TRBV11-2	TRBJ2-5	1.3	CD8T	24
	7	ASSRNPYDTYEQY	TRBV6-5	TRBJ2-7	1.9	CD8T	738	7	ASSPPVDRVVEKLF	TRBV7-9	TRBJ1-4	1.2	CD8T	57
	8	ASSNTGTGNTGELF	TRBV7-9	TRBJ2-2	1.8	Tax-CTL	3	8	ASSPWAEGNTIY	TRBV9	TRBJ1-3	1.0	CD8T	19
	9	ASSPRTGGNEQF	TRBV6-4	TRBJ2-1	1.5	UI		9	ASTPASGGIYNEQF	TRBV5-1	TRBJ2-1	1.0	CD8T	9
	10	ASSRGTGYYEQY	TRBV7-8	TRBJ2-7	1.4	UI		10	ASSFTPEAQY	TRBV6-5	TRBJ2-5	0.8	CD8T	135
	11	SVESVREAF	TRBV29-1	TRBJ1-1	1.4	UI		11	ASSLEFPDTQY	TRBV7-6	TRBJ2-3	0.7	CD8T	39
	12	ASSPRTGDAF	TRBV19	TRBJ1-1	1.4	UI		12	ASSEDREATIY	TRBV2	TRBJ1-3	0.6	UI	
	13	ASMETNAYEQY	TRBV19	TRBJ2-7	1.4	UI		13	ASSLAGRGEQY	TRBV11-1	TRBJ2-7	0.6	UI	
	14	ASSHQNTEAF	TRBV5-4	TRBJ1-1	1.4	CD8T	13	14	SVENTDTQY	TRBV29-1	TRBJ2-3	0.6	UI	
	15	ASSSTGDTQY	TRBV5-4	TRBJ2-3	1.3	UI		15	AWMTGLPPYEQY	TRBV30	TRBJ2-7	0.6	UI	
	16	ASKVGQYPNYGYT	TRBV19	TRBJ1-2	1.1	UI		16	ASRR **DR** SYEQY	TRBV6-1	TRBJ2-7	0.6	Tax-CTL	3
	17	SVDGGVGETQY	TRBV29-1	TRBJ2-5	1.1	CD8T	102	17	ASSVDLADTQY	TRBV2	TRBJ2-3	0.5	UI	
	18	ASSDRPEQNTIY	TRBV9	TRBJ1-3	1.0	UI		18	ASSGAPGGEQF	TRBV10-2	TRBJ2-1	0.5	UI	
	19	SVDYWTSGGLTDTQY	TRBV29-1	TRBJ2-3	0.9	CD8T	72	19	ASSEMTAYQETQY	TRBV2	TRBJ2-5	0.5	CD8T	12
	20	ASSYSSSGTENYGYT	TRBV6-6	TRBJ1-2	0.9	UI		20	SVVLTGGATEAF	TRBV29-1	TRBJ1-1	0.5	CD8T	1087
	21	AISVGSNTEAF	TRBV10-3	TRBJ1-1	0.9	UI		21	SVERDRDTQY	TRBV29-1	TRBJ2-3	0.4	UI	
	22	ASSVEGKPTDTQY	TRBV2	TRBJ2-3	0.9	UI		22	ARSRGAEDTQY	TRBV30	TRBJ2-3	0.4	UI	
	23	SARGRETQY	TRBV29-1	TRBJ2-5	0.8	UI		23	ATSDRTRLFEDTQY	TRBV24-1	TRBJ2-3	0.4	Tax-CTL	4
	24	ASTPGQTFQETQY	TRBV6-5	TRBJ2-5	0.8	UI		24	ASSRDSGRLGQPQH	TRBV5-5	TRBJ1-5	0.4	CD8T	1444
	25	ASSLSGEDEPQH	TRBV12-3	TRBJ1-5	0.8	UI		25	ASSSSSANYGYT	TRBV7-9	TRBJ1-2	0.4	CD8T	34
	26	SVPEGKRNGEQF	TRBV29-1	TRBJ2-1	0.8	UI		26	SATYGTNQPQH	TRBV20-1	TRBJ1-5	0.4	UI	
	27	ASRDRSGGLGTDTQY	TRBV28	TRBJ2-3	0.8	UI		27	ASSLGQSSYNEQF	TRBV5-1	TRBJ2-1	0.4	UI	
	28	SVGEGNQPQH	TRBV29-1	TRBJ1-5	0.8	UI		28	ACYRVAGSSYEQY	TRBV30	TRBJ2-7	0.4	UI	
	29	ASSIGLGTHYGYT	TRBV19	TRBJ1-2	0.7	UI		29	SVGMDGLEQY	TRBV29-1	TRBJ2-7	0.4	UI	
	30	ASSSAGVTGELF	TRBV7-6	TRBJ2-2	0.7	CD8T	8	30	ASSFRALPRNEQF	TRBV9	TRBJ2-1	0.4	UI	

TCRß CDR3 amino acid (AA)-sequences of top 30 T-cell clones in the CSF of four each HAM patient (HAM-8, -9, -11 and -12) analyzed by NGS illumina Miseq. We identified a total of 1,428 T-cell clones (HAM-8), 906 (HAM-9), 6,207 (HAM-11), and 3,002 T-cell clones (HAM-12) in the CSF samples, respectively. The belonging of T-cell clones in the CSF was conducted by comparing the TCR repertoires of CD8^+^ T-cells and Tax_301-309_-CTLs in PB, respectively. CSF neopterin is a HAM disease activity biomarker (32, 33). Entries that are in bold and underlined indicate the conserved CDR3 AA sequences, which is "PDR", or second-major AA-sequence motifs ("P-R", "PD-", and "-DR") in TCRß CDR3 of each Tax_301-309_-CTL clone. (%) indicates the frequencies of each clone in the CSF. UI, unidentified. Entries that are in bold and underlined indicate the conserved CDR3 AA sequences, which is "PDR", or second-major AA-sequence motifs ("P-R", "PD-", and "-DR") in TCRß CDR3 of each Tax301-309-CTL clone.

To speculate on the efficiency of migration and accumulation of CD8^+^ T-cells and Tax_301-309_-CTLs at the clone levels in the CSF, their clonal rankings were compared between PB and CSF (**Figure 3B**). Although the clonal rankings of CD8^+^ T-cells and Tax_301-309_-CTL were not constantly parallel between PB and CSF, Tax_301-309_-CTL clones that further clonally expanded after infiltrating the CSF from PB were observed more frequently in the two patients (HAM-9 and-11) with high levels of inflammation (CSF neopterin, ≥31 pmol/ml, **Table 1**) than in the two patients (HAM-8 and -12) with moderate inflammation levels (CSF neopterin, ≥17 pmol/ml, **Table 1**). Notably, in HAM-9 with high levels of inflammation, one PDR^+^Tax_301-309_-CTL clone, although very rare in PB (<0.001% of Tax_301-309_-CTLs), rapidly clonally expanded after infiltrating the CSF, reaching a high rank of 30th among CSF T-cell clones.

### Inflammatory status and the frequency of Tax_301-309_-CTLs with unique TCRs in the CSF of HAM patients

We have previously reported that CSF CXCL10 and neopterin were strongly correlated with the rate of disease progression in HAM (31, 32). Here, to assess whether infiltrating Tax_301-309_-CTLs expressing unique TCR-motif PDR, or (-DR, P-R, and PD-) would be linked to the promotion of CNS inflammation of HAM, we evaluated the relationship between their frequencies in PB and CSF and the CSF levels of CXCL10 and neopterin.

**Figure 4:**
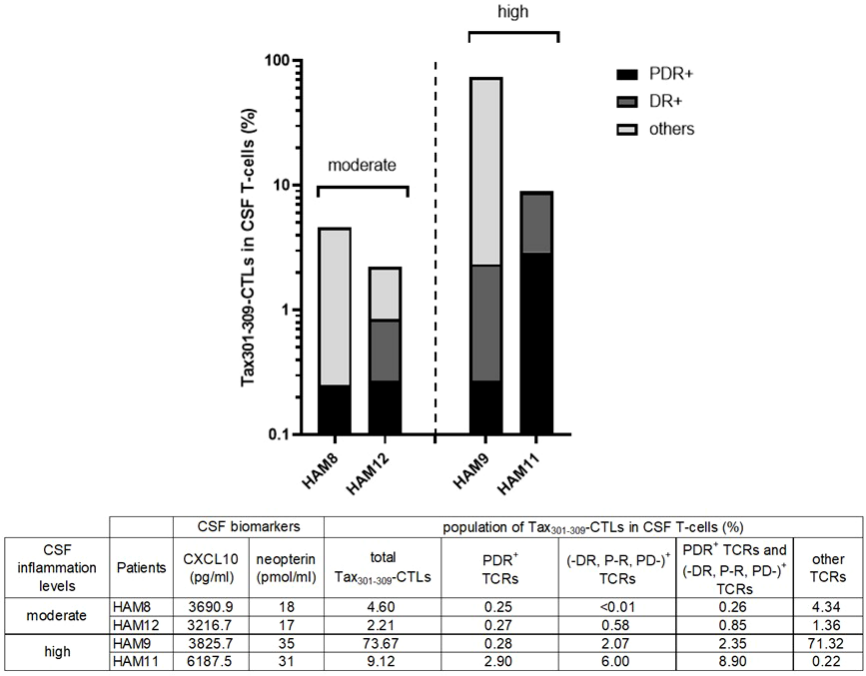
The frequencies of Tax_301-309_-CTLs expressing the unique TCR motifs in the CSF and the inflammation status of HAM patients The graph shows the frequencies of the total Tax_301-309_-CTLs and Tax_301-309_-CTLs expressing unique TCR-motifs (PDR or -DR, P-R, PD-) in the CSF of the HAM patients with the moderate (HAM-8 and -12) or high (HAM-9 and -11) levels of CSF inflammation markers (CXCL10 and neopterin).

As a result, there was no clear correlation between the frequencies of Tax_301-309_-CTLs expressing unique TCR-motif PDR or (-DR, P-R, PD-) in PB and the CSF levels of CXCL10 and neopterin (**Supplementary Figure 1**). However, as shown in **Figure 4**, Tax_301-309_-CTLs expressing unique TCR-motif PDR or (-DR, P-R, PD-) were 10-fold more abundant in the CSF of the two patients (HAM-9 and-11) with high levels of inflammation (CSF neopterin, ≥31 pmol/ml) compared to the two patients (HAM-8 and -12) with moderate inflammation levels (CSF neopterin, ≥17 pmol/ml). Specifically, in HAM-11, a patient with high levels of inflammation, a high frequency of PDR^+^Tax_301-309_-CTLs (2.9% of total CSF T-cells) was found in the CSF. Thus, Tax_301-309_-CTLs expressing unique TCR-motif PDR or (-DR, P-R, PD-) were frequently observed in the CSF of HAM patients with inflammation, and the frequency of them in the CSF rather than PB may better reflect the CNS inflammation of HAM patients.

### Single-cell RNA sequence of Tax_301-309_-CTLs with unique TCRs of HAM patients

**Figure 5:**
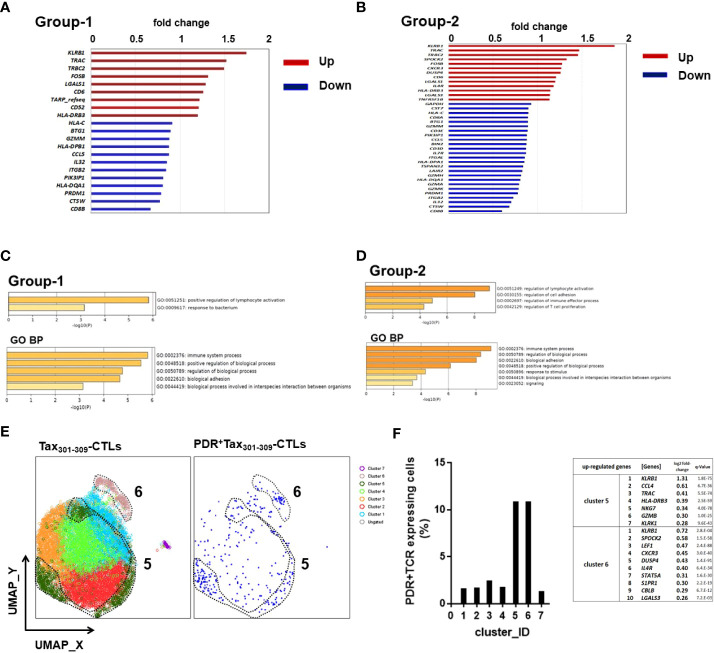
scRNA-seq profiling of Tax_301-309_-CTLs expressing the unique TCR motifs in PBMCs of HAM patients We performed scRNA-seq analysis for Tax_301-309_-CTLs from three HAM patients focusing on the two groups, group-1: Tax_301-309_-CTLs expressing PDR-motif (PDR^+^Tax_301-309_-CTLs) and group-2: sum of Tax_301-309_-CTLs expressing PDR-motif and (-DR, P-R, and PD-)-motif. The DEG analysis was performed for **(A)** group-1 and **(B)** group-2, respectively. GO function and pathway enrichment analysis was performed for the up-regulated genes in **(C)** group-1 and **(D)** group-2, respectively. BP: the biological process of GO category. **(E)** cell clustering of Tax_301-309_-CTLs with UMAP plot and overlay of PDR^+^Tax_301-309_-CTLs. Consequently, seven clusters were formed in the Tax_301-309_-CTL population. **(F)** PDR^+^Tax_301-309_-CTLs were concentrated in both clusters 5 and 6 and the genes upregulated in the corresponding clusters are shown.

To further understand the potential function of Tax_301-309_-CTLs expressing unique TCR motifs (PDR or -DR, P-R, PD-), we performed scRNA-seq on FACS-sorted Tax_301-309_-CTLs in PBMCs of HAM patients (**Figure 5**). The data from a total of 11,029 Tax_301-309_-CTLs (HAM-1: 1,414 cells, HAM-7: 9,290 cells, and HAM-8: 325 cells, respectively) was supplied to be processed in the DEG analysis and in the Seurat package to perform downstream clustering of the cells. In DEG analysis, we focused on the two groups in Tax_301-309_-CTLs. Group-1 was a population of PDR^+^Tax_301-309_-CTLs (336 cells) and group-2 was a population of the sum of Tax_301-309_-CTLs expressing PDR or (-DR, P-R, and PD-)-motif (453 cells). DEG analysis indicated that 9 genes were identified as up-regulated genes in group-1 (**Figure 5A**). Particularly, natural killer (NK) gene *KLRB1* (CD161), T-cell receptors *TRAC* (TCR-α), and *TRBC2* (TCR-ß) were upregulated approximately more than 1.5-fold compared to Tax_301-309_-CTLs expressing other repertoires. In group-2, 13 genes were identified as up-regulated genes (**Figure 5B**) and *KLRB1* (CD161), *TRAC* (TCRα), and *TRBC2* (TCR-ß) were again approximately more than 1.5-fold compared to Tax_301-309_-CTLs expressing other repertoires (**Supplementary Table 3**). Furthermore, analysis of enriched GO functions of up-regulated genes of groups-1 and -2 was examined using the Metascape database platform, respectively (**Figures 5C, D**). As a result, GO indicated that the main pathway was (positive) regulation of lymphocyte activation in both groups-1 and -2. Moreover, GO biological processes of both groups-1 and -2 were most enriched in the immune system process.

Finally, to further understand the potential function of Tax_301-309_-CTLs expressing unique TCR motif, especially on shared TCR-motif PDR (cells in group-1), cell clustering of Tax_301-309_-CTLs was performed using UMAP plots and individual PDR^+^Tax_301-309_-CTLs were representatively overlaid on the plots (**Figure 5E**). As a result, seven major cell clusters (clusters 1-7) were identified from Tax_301-309_-CTLs, and PDR^+^Tax_301-309_-CTLs were concentrated in clusters 5 and 6, respectively, constituting approximately 10% of cells in each cluster (**Figure 5F**). Notably, *KLRB1* gene expression was selectively highest in both clusters 5 and 6, whereas it was unidentified in the other clusters (**Supplementary Table 4**), corresponding to the results of upregulated genes in DEGs of group-1 of PDR^+^Tax_301-309_-CTLs (**Figure 5A**). Upregulation of *TRAC* and *TRBC2* genes in the DEG analysis did not match the results of clusters 5 and 6, respectively.

Thus, scRNA-seq for Tax_301-309_-CTLs indicated that the up-regulated genes in Tax_301-309_-CTLs expressing PDR or (-DR, P-R, and PD-)-motifs may be associated with the immune system process of T-cell activation, and the shared PDR^+^Tax_301-309_-CTLs among HTLV-1-infected individuals might be activated in association with upregulation of *KLRB1* gene expression.

## Discussion

After development of NGS-based TCR repertoire analysis technology, studies are accumulating data on shared (public) TCRs in infectious diseases, malignancy, and autoimmunity (31, 33–37). In the present study, we also comprehensively analyzed Tax_301-309_-specific TCR repertoires of HLA-A*24:02^+^ HAM patients by NGS sequencing and found that they were skewed with a preference for unique TCR AA-sequence PDR- or (-DR, P-R, and PD-), regardless of disease duration and inflammation status of HAM. Based on the comprehensive evaluation of the TCR repertoires of Tax_301-309_-CTLs in HAM patients in the present study and those in ACs and ATL patients previously analyzed (13, 14), we confirmed that PDR is a shared (public) TCR-motif for the HTLV-1 Tax_301-309_ epitope among HLA-A*24:02^+^ HTLV-1-infected individuals. Regarding HTLV-1 Tax_11-19_-specific TCRs which are restricted by HLA-A*02:01, it has been demonstrated that AA-sequence (PG-G) in the TCR-ß CDR3 may be conserved among Tax_11-19_-specific T-cells (38) and the sequence was observed in the muscle biopsies obtained from a patient with HLA-A*02:01^+^ HAM (39).

In chronic viral infections, T_SCM_ is thought to play a central role in the maintenance of long-term human T-cell immunity by reconstituting the entire spectrum of memory and effector T-cell subsets (28–30, 40). In HTLV-1 infections, a study has reported the frequency of T_SCM_ of CD8^+^ T-cells increased in HAM patients compared to healthy volunteer (41). In the present study, our data showed that T_SCM_ of Tax_301-309_-CTLs in PB of HAM patients were decreased compared to ACs (**Figure 1E**), although the absolute frequency of Tax_301-309_-CTLs with the predominant T_EM_ phenotype were increased in PB compared to ACs (**Figure 1B**). In fact, we observed no clear positive correlation between the absolute frequencies of T_SCM_ and T_EM_ of Tax_301-309_-CTLs in PB of HAM patients (data not shown). These results imply that the abundant memory Tax-CTLs in PB of HAM patients compared to ACs would be more likely to be due to clonal expansion of Tax-CTLs with highly activity potential against HTLV-1 (42, 43), rather than due to the reconstitution by T_SCM_ of Tax-CTLs after the onset of HAM.

Previous studies have demonstrated accumulation of HTLV-1-infected cells and Tax-CTLs infiltrating the CSF of HAM patients (19, 20). In one study, the visualization of Tax-CTLs in the spinal cord of HAM patients using Tax-tetramer staining directly demonstrated that the frequency of Tax-CTLs was more than 20% of CD8^+^ cells infiltrating the CNS (44). Furthermore, recently, Nozuma et al. revealed that an AA-sequence motif (PGLAG) was conserved in the TCR-ß CDR3 of Tax_11-19_–specific CD8^+^ T-cells among HLA-A*02:01^+^ HAM patients and expanded HTLV-1 Tax_11-19_–specific CD8^+^ T-cell clones in PB were also enriched in the CSF of the same patient by NGS-based TCR repertoire analysis technology (37). In the present study, we also showed the clonal dynamics of CD8^+^ T-cells and Tax_301-309_-CTLs before and after CSF infiltration by simultaneous analysis of the TCR repertoire of PB and CSF samples from the same HAM patients. Our data indicated that Tax_301-309_-CTL clones expressing PDR or (-DR, P-R, PD-)-motif were more frequently observed in the CSF of HAM patients with severe inflammation compared to that of patients with moderate inflammation. Importantly, a patient with severe inflammation demonstrated a dramatic clonal expansion of one PDR^+^Tax_301-309_-CTL clone after infiltrating the CSF from PB. Our findings supported the hypothesis regarding the potential role of PDR^+^Tax_301-309_-CTLs to promote inflammation in the CNS of HAM. It is still unclear whether there is a mechanism by which Tax_301-309_-CTLs, particularly PDR^+^Tax_301-309_-CTLs, selectively migrate to the CSF, because we failed to find any obvious factors associated with T-cell migration by scRNA-seq for PDR^+^Tax_301-309_-CTLs using T-cell expression gene panel.

Recent scRNA-seq technology has been used as a powerful tool to reveal cellular heterogeneity and discover new cell types in various human diseases (24, 45, 46). Since Tax_301-309_-CTLs in HAM patients potentially react to the same Tax_301-309_ epitope and its population was relatively homogeneous (most cells were effector memory T-cells), it seemed difficult to profile PDR^+^Tax_301-309_-CTLs by scRNA-seq. Interestingly, however, the scRNA-seq indicated that at least *KLRB1* could be a gene expression signature of PDR^+^Tax_301-309-_CTLs. The role of the expression of NK cell markers including CD161 (gene: *KLRB1*) on human antigen-specific CD8^+^ T-cells has been under investigation by several groups (47–50). Previous studies reported that CD161 was preferentially expressed on human memory T-cell subsets (48, 49) and these cells showed highly cytotoxic potential, long life, and drug-effluxion (47, 50), although the signaling cascade of events that lead to the effector functions is poorly understood. Unfortunately, in the present study, we could not approach the signal pathway of *KLRB1* expression in PDR^+^ Tax_301-309_-CTLs. Mathewson et al. recently revealed that glioma-infiltrating CD8^+^ T-cells with high cytotoxicity expressed several NK cell markers, including *KLRB1* (CD161) by scRNA-seq (51). Thus, these data from scRNA-seq and our accumulating function data of PDR^+^Tax_301-309_-CTLs in *in vitro* (13–16) and *in vivo* (52) experiments support the potential role of PDR^+^Tax_301-309_-CTLs to promote CNS inflammation of the patients with HAM. Since gene enrichment by scRNA-seq does not always reflect protein expression on cell surface (45), we plan to confirm the CD161 expression on PDR^+^Tax_301-309_-CTLs and discuss their highly cytotoxic potential in relation to CD161 signaling events in future study.

The present study provides a better understanding of HTLV-1-specific CTLs shared among HLA-A*24:02^+^ HTLV-1-infected individuals under the inflammatory pathogenesis of HAM. Further studies on a larger scale are needed, before we can reach a definitive conclusion regarding the strength of the biological impact of PDR^+^Tax_301-309_-CTLs on promoting inflammation within the CNS lesions of HAM. If confirmed, however, this would offer an interesting insight as regulating the inflammation of HLA-A*24:02^+^ HAM, and the PDR^+^Tax_301-309_-CTLs may serve as a candidate target to ameliorate the inflammatory cascade in HLA-A*24:02^+^ HAM.

## Data availability statement

The datasets presented in this study are included in the article/**Supplementary Material**. scRNA-seq datasets can be found in online repositories, GSE210786 (GEO). Further inquiries can be directed to the corresponding authors.

## Ethics statement

The studies involving human participants were reviewed and approved by the Institutional Review Boards of St. Marianna University School of Medicine (#1646) and the Institute of Medical Science, The University of Tokyo (30-4-B0501). The patients/participants provided their written informed consent to participate in this study.

## Author contributions

YT designed the study, performed experiments, analyzed data, and wrote the manuscript. TS, MN, YoK, TM, and YY conducted the study and contributed to the discussion and wrote the manuscript. KU collected AC samples and clinical data and gave his advice about the experimental procedures. NY, JY, NA, and SA collected samples and clinical data. KT and YaK performed the experiment using CSF samples. All authors contributed to the article and approved the submitted version.

## Funding

This work was supported by JSPS KAKENHI Grant Number JP22H04923 (CoBiA). A grant from the Practical Research Project for Rare/Intractable Diseases of the Japan Agency for Medical Research and Development (No. JP22ek0109529), a grant from Rare and Intractable Diseases from the Ministry of Health, Labour and Welfare of Japan (No. JPMH22FC1013), and a grant from Japan Society for the Promotion of Science (JSPS) KAKENHI (No. JP22H02987) for YY and a grant from JSPS KAKENHI (No. JP22K07513) and a grant from Takeda Science Foundation for YT.

## Acknowledgments

We thank Erika Horibe and Kiyomi Kubo at the Institute of Medical Science, The University of Tokyo for their assistance with collecting samples and clinical data. Akira Nishimura at the Department of Pediatrics and Developmental Biology, Tokyo Medical and Dental University (TMDU) provided support in single-cell RNA-seq data analysis.

## Conflict of interest

SA is employed by LSI Medience Corporation.

The remaining authors declare that the research was conducted in the absence of any commercial or financial relationships that could be construed as a potential conflict of interest.

## Publisher’s note

All claims expressed in this article are solely those of the authors and do not necessarily represent those of their affiliated organizations, or those of the publisher, the editors and the reviewers. Any product that may be evaluated in this article, or claim that may be made by its manufacturer, is not guaranteed or endorsed by the publisher.

## Supplementary material

SUPPLEMENTARY Figure 1 Correlation between the frequencies of Tax301-309-CTLs expressingunique TCR-motif in PB and the CSF levels of CXCL10 and neopterinCorrelation were tested by Spearman’s rank correlation test. p-values,0.05 were considered statistically significant.
