# Supplementary figures and images for: Potential role of HTLV-1 Tax-specific cytotoxic t lymphocytes expressing a unique t-cell receptor to promote inflammation of the central nervous system in myelopathy associated with HTLV-1
Source: Front Immunol. 2022 Aug 23;13:993025. doi: 10.3389/fimmu.2022.993025 (PMC9446235; doi:10.3389/fimmu.2022.993025)

# Supplementary Figure 1

CSF CXCL10

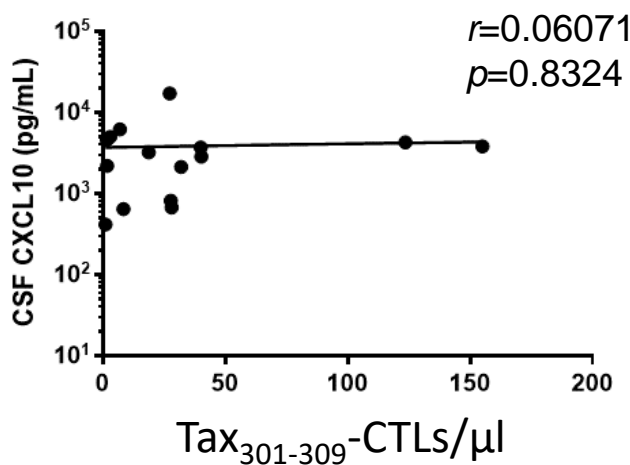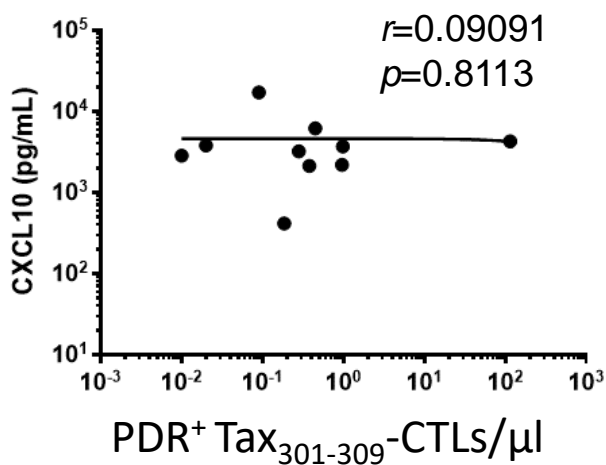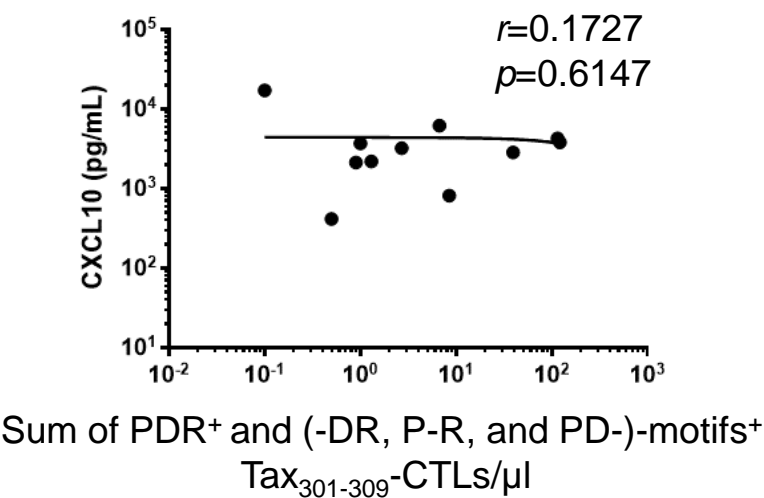

CSF neopterin

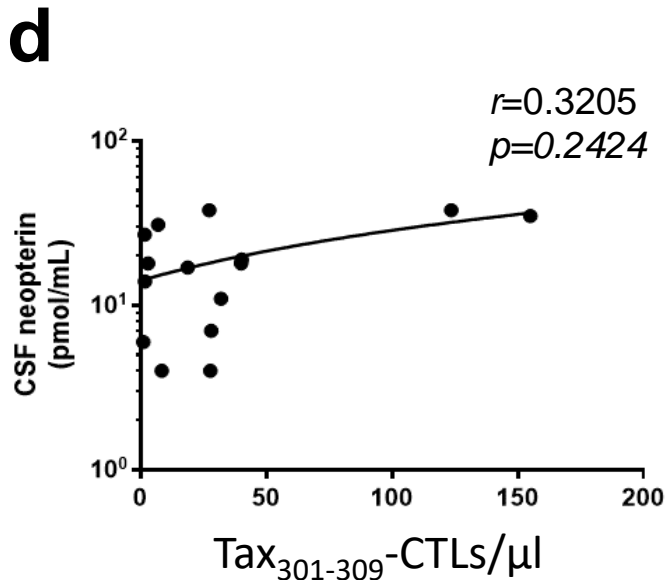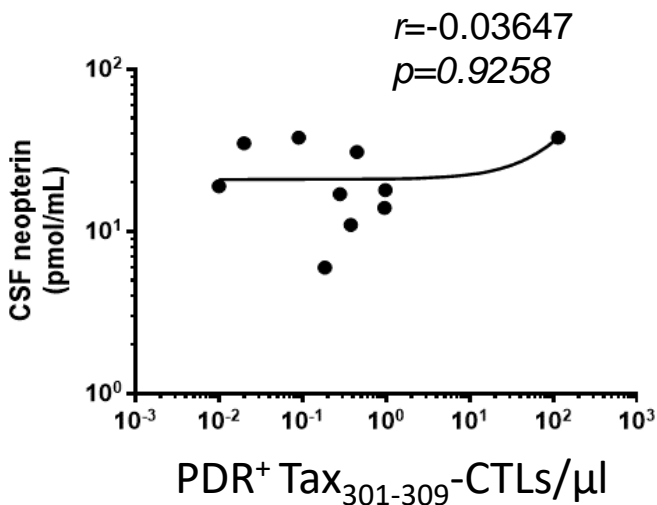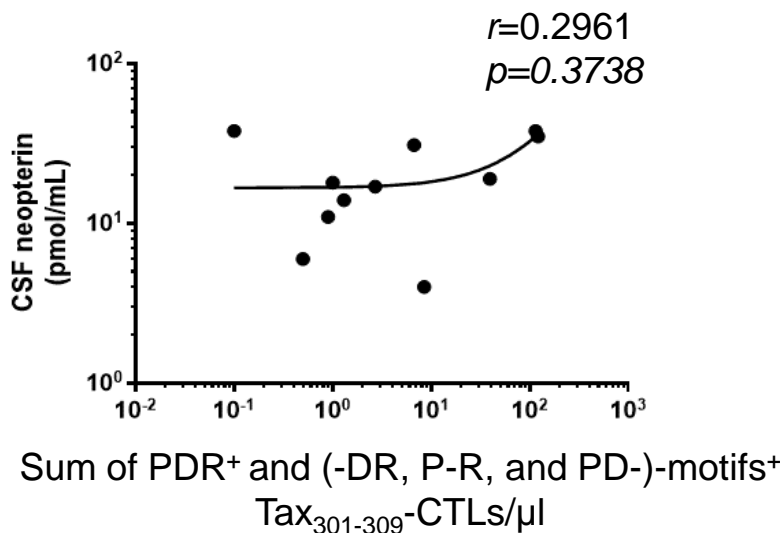

Supplement: SUPPLEMENTARY Figure 1 — Correlation between the frequencies of Tax301-309-CTLs expressingunique TCR-motif in PB and the CSF levels of CXCL10 and neopterinCorrelation were tested by Spearman’s rank correlation test. p-values,0.05 were considered statistically significant. [file Presentation_1.pdf]
